# Intraoperative fluoroscopic protocol to avoid rotational malalignment after nailing of tibia shaft fractures: introduction of the ‘C-Arm Rotational View (CARV)’

**DOI:** 10.1007/s00068-022-02038-2

**Published:** 2022-07-30

**Authors:** Nils Jan Bleeker, Job N. Doornberg, Kaj ten Duis, Mostafa El Moumni, Inge H. F. Reininga, Ruurd L. Jaarsma, Frank F. A. IJpma, On behalf of the Traumaplatform 3D Consortium, L. M. Goedhart, B. de Cort, L. A. M. Hendrickx, M. ter Horst, J. Gorter, R. J. van Luit, P. Nieboer, W. Füssenich, T. Zwerver, R. Koster, J. J. Valk, L. Reinke, J. G. Bleeker, M. Cain, F. J. P. Beeres, G. M. M. J. Kerkhoffs

**Affiliations:** 1 Department of Orthopaedic Trauma Surgery, University Medical Center Groningen, University of Groningen, Groningen, The Netherlands; 2 Department of Orthopaedic Trauma Surgery, Flinders Medical Center and Flinders University, Adelaide, Australia

**Keywords:** Intramedullary-nailing, Tibia shaft fractures, Rotational malalignment, ‘C-Arm Rotational View (CARV)’

## Abstract

**Purpose:**

Rotational malalignment (≥ 10°) is a frequent pitfall of intramedullary-nailing of tibial shaft fractures. This study aimed to develop an intraoperative fluoroscopy protocol, coined ‘C-Arm Rotational View (CARV)’, to significantly reduce the risk for rotational malalignment and to test its clinical feasibility.

**Methods:**

A cadaver and clinical feasibility study was conducted to develop the CARV-technique, that included a standardized intraoperative fluoroscopy sequence of predefined landmarks on the uninjured and injured leg in which the rotation of the C-arm was used to verify for rotational alignment. A mid-shaft tibia fracture was simulated in a cadaver and an unlocked intramedullary-nail was inserted. Random degrees of rotational malalignment were applied using a hand-held goniometer via reference wires at the fracture site. Ten surgeons, blinded for the applied rotation, performed rotational corrections according to (1) current clinical practice after single-leg and dual-leg draping, and (2) according to the CARV-protocol. The primary outcome measure was the accuracy of the corrections relative to neutral tibial alignment. The CARV-protocol was tested in a small clinical cohort.

**Results:**

In total, 180 rotational corrections were performed by 10 surgeons. Correction according to clinical practice using single-leg and dual-leg draping resulted in a median difference of, respectively, 10.0° (IQR 5.0°) and 10.0° (IQR 5.0°) relative to neutral alignment. Single-leg and dual-leg draping resulted in malalignment (≥10°) in, respectively, 67% and 58% of the corrections. Standardized correction using the CARV resulted in a median difference of 5.0° (IQR 5.0°) relative to neutral alignment, with only 12% categorized as malalignment (≥10°). The incidence of rotational malalignment after application of the CARV decreased from 67% and 58% to 12% (*p* =  <0.001). Both consultants and residents successfully applied the CARV-protocol. Finally, three clinical patients with a tibial shaft fracture were treated according to the CARV-protocol, resulting all in acceptable alignment (<10°) based on postoperative CT-measurements.

**Conclusion:**

This study introduces an easy-to-use and clinically feasible standardized intraoperative fluoroscopy protocol coined ‘C-arm rotational view (CARV)’ to minimize the risk for rotational malalignment following intramedullary-nailing of tibial shaft fractures.

**Supplementary Information:**

The online version contains supplementary material available at 10.1007/s00068-022-02038-2.

## Introduction

Rotational malalignment remains an iatrogenic pitfall of intramedullary-nailing of tibial shaft fractures which occurs in up to 30% of the cases [1–7]. Alignment control is challenging and visual assessment for adequate alignment is difficult as tibia fractures are often accompanied by soft tissue injury, swelling, and various respective positions of the leg on the operating table [7]. Furthermore, the use of closed reduction techniques and presence of multiple fracture fragments may complicate adequate alignment and interpretation of fluoroscopy images [8].

Rotational malalignment is defined as a rotation ≥10° if compared to the contralateral side [1–9] and may lead to functional impairments [10–14]. Patients with rotational malalignment qualify for monetary compensation according to the “Guides to the Evaluation of Permanent Impairment” [15]. Knowledge on intraoperative identification and how to avoid rotational malalignment is therefore of paramount importance. However, studies on how to avoid rotational malalignment during intramedullary-nailing are scarce [16–19]. Postoperatively, low-dose CT-assessment is considered the gold standard and allows for early detection and revision of rotational errors [7, 8]. Hence, CT-assessment is performed postoperatively in case of a clinical suspicion when the opportunity for direct revision has passed. In contrast, for patients with femoral shaft fractures, multiple studies described various intraoperative fluoroscopic strategies and protocols to avoid rotational malalignment during intramedullary-nailing [20–24]. However, an intraoperative fluoroscopic protocol to avoid rotational malalignment during intramedullary-nailing following tibia shaft fractures is still lacking.

The purpose of this cadaveric study was to develop an easy-to-use intraoperative fluoroscopy protocol, coined *‘*C-Arm Rotational View (CARV)’, using a standard C-arm image-intensifier in order to reduce the risk of rotational errors during intramedullary-nailing of tibia fractures. The following primary research question was posed: can we improve the accuracy of rotational alignment during intramedullary-nailing of tibia shaft fractures utilizing our CARV-protocol relative to present clinical standards?

## Materials and methods

A cadaver study was performed in the Skills Center of the University Medical Center Groningen, the Netherlands between March 2021 and July 2021. Fresh-frozen cadaveric specimens were used and CT scans of both extremities confirmed that there were no preexistent physiological rotational differences between the left and right tibia. Ten orthopedic trauma surgeons (five residents, five consultants) participated in this study. A standard C-arm image-intensifier (GE (General Electric) OEC 9800, Salt Lake City, USA) was used to obtain fluoroscopic images. The total exposure of radiation during the experiment ranged between 0.001 and 0.003 mSv. Approval of the Medical Ethic Review Board of the University Medical Centers Groningen was obtained with number 201900721 in accordance to the Declaration of Helsinki.

### Research setting

**Fig. 1:**
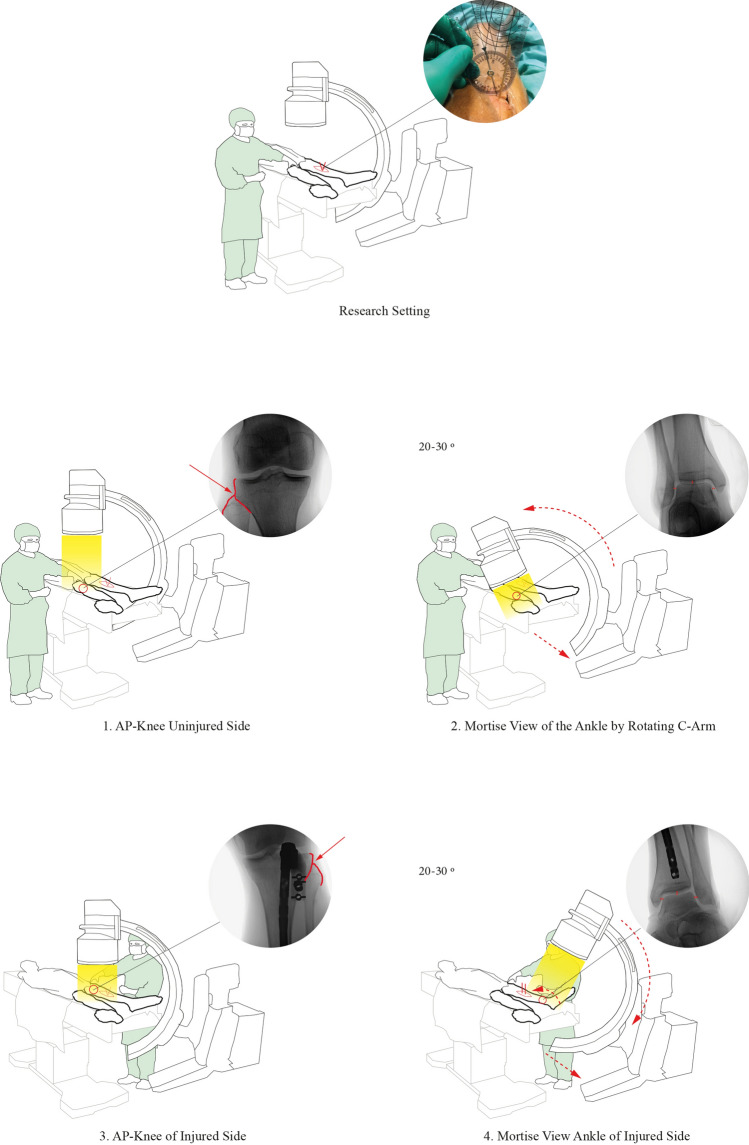
Schematic overview of the “C-arm Rotational View (CARV)”. The CARV is an intraoperative fluoroscopy sequence comparing the uninjured and injured leg in which the degree of rotation of the C-arm itself is used to correct for rotational malalignment of the tibia. At the uninjured leg, a perfect mortise-view is taken with the knee in AP-position by rotating the C-arm between 20 and 30°. At the injured leg, the C-arm is rotated to the same extent in the opposite direction while the knee is in AP-position and subsequently the distal part of the lower leg is rotated until a perfect mortise-view is achieved, indicating symmetrical tibial alignment.

In this cadaver study, a 4 cm longitudinal incision was made at the mid-shaft of the tibia and a transverse tibia fracture and same-height fibula fracture was created with an osteotomy. An unlocked intramedullary-nail (Expert Tibial Nail; DePuy Synthes, Switzerland) was introduced. The unlocked nail press-fitted the isthmus, allowing to rotate the distal tibia over the nail and to apply various degrees of internal and external rotation. Two parallel wooden reference wires were placed on each side of the fracture in order to measure the rotation-angle with a hand-held goniometer (Fig. 1). The fracture and reference wires were covered with a sterile drape in order to blind the observers to the wire positions.

Rotation of 0° to 30°—both internal and external—were applied by NJB and FIJ. Statistical software package SPSS was used to generate a randomized sequence of applied rotations in order to correct for possible learning effects. The observer, blinded to the applied rotation, first had to estimate the degree of rotation. Hereafter, the observer had to perform a rotational correction by rotating the distal tibia over the nail until the observer was convinced that neutral alignment was obtained. The tibia was fixed in this position and the reference wires were only exposed to the investigators by removing the sterile drape. The angle between the reference wires was measured and recorded. The rotational corrections were first performed according to current clinical practice (as described in the next paragraph) and second using the CARV-protocol (as described in the subsequent paragraph).

### How accurate can surgeons estimate and correct tibia (mal)rotation according to current clinical practice?

Present clinical standards included first estimation and correction of tibia (mal)rotation with only the injured leg exposed (single-leg draping) and second estimation and correction of tibia (mal)rotation with two legs being exposed (dual-leg draping). Correction techniques were according to the surgeons’ preferences and included clinical assessment of the position of the leg, palpating the anteromedial rim of the tibia, fluoroscopy assessment of the cortical width at the fracture site, or a combination of techniques.

### Can we improve the accuracy of rotational alignment by use of the CARV-protocol?

**Fig. 2:**
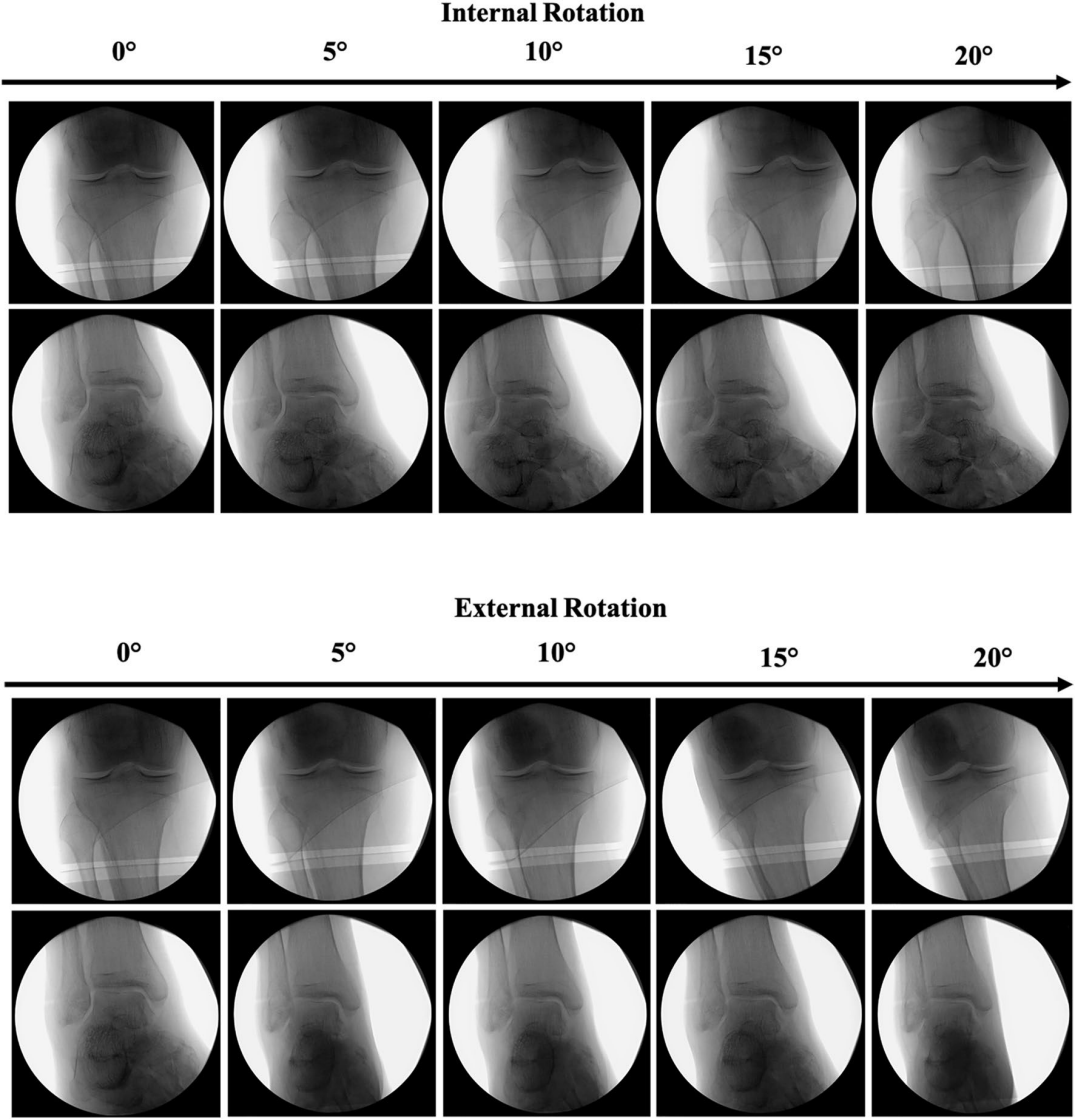
Schematic overview of alterations of the proximal and distal landmarks of the CARV-protocol after applying different degrees of both internal and external rotations. **a** After application of internal rotation, the proximal fibula head is more exposed while the medial clear space of the ankle joint minimizes. **b** After application of external rotation, there was an increased superimposement of the proximal fibula head by the lateral tibia plateau with lesser distance of the lateral clear space of the ankle joint.

The CARV-technique is a standardized intraoperative fluoroscopic algorithm in which an anteroposterior (AP)-view of the knee at the contralateral side is combined with a perfect mortise-view of the ankle, obtained by rotating the C-arm without manipulating the ankle. The standardized fluoroscopic views at the contralateral side combined with rotation of the C-arm itself were used as an example to align the tibia at the injured side (Fig. 2a, b). The detailed workflow of CARV includes (Fig. 1): 

1. Determination of the fluoroscopy landmarks at the contralateral side. First, the C-arm was positioned in neutral position (0°) and an AP-view of the knee was obtained, defined by the exact intersection of the lateral cortex of the proximal tibia trough the tip of the proximal fibula (Fig. 1a) [25]. Hereafter, the C-arm shifted to the ankle while the surgeon kept the knee in exact AP-position (Fig. 1b). A mortise-view was obtained by rotating the C-arm 20–30° without manipulating the ankle. The mortise-view was defined as an AP-view of the ankle joint with equal medial, lateral and superior clear spaces [26, 27] (Fig. 1b). Both images were saved and converted to the output window of the C-arm. The degree of rotation of the C-arm was recorded.
2. Determination of the fluoroscopy landmarks of the injured side. An identical AP-knee as compared to the contralateral side was obtained (Fig. 1c). Subsequently, a mortise-view of the ipsilateral ankle was obtained by rotating the C-arm in the exact opposite position as determined on the opposite side (Fig. 1b, Fig. 1d). In case of an adequate tibial alignment, the mortise-view should appear and be identical to the contralateral reference mortise-view of the ankle. Any discrepancies indicated rotational malalignment and subsequent correction was performed by rotating the distal tibia over the nail while the knee remained in AP-position until and identical mortise-view was established, indicating neutral alignment (Fig. 1d).

### Definition of outcome measures

Ten observers performed a total of 180 rotational corrections (60 unstandardized corrections after single-leg draping; 60 unstandardized corrections after dual-leg draping; 60 corrections according to the standardized CARV-technique). The primary outcome was the accuracy of rotational correction, assessed with a hand-held goniometer and measured in degrees at the level of the tibia fracture (Fig. 1). Neutral alignment of the tibia was defined as an angle of 0° between the reference wires at the level of the tibia fracture as compared to the contralateral reference. The results were categorized into acceptable (<10°) and unacceptable (≥10°) rotational alignment.

### Clinical feasibility

The clinical feasibility of the CARV-protocol was tested in a small prospective cohort representing a total of three patients treated with intramedullary-nailing for a tibial shaft fracture (supplementary materials). Patients were positioned in supine position and both extremities were draped free. No traction was applied. Rotational correction was performed using the standardized CARV-protocol after insertion of the unlocked intramedullary-nail. After obtaining the correct predefined fluoroscopy landmarks, definitive locking was performed. All patients underwent postoperative CT-assessment in order to verify the alignment of the tibia according to our standardized measurement technique [8].

### Statistical analyses

Statistical software package SPSS 28.0 was used for analyzing data. The rotational accuracy was presented as median with interquartile range (IQR). The differences in rotational accuracy between CARV and present clinical standards were calculated by use of the Mann–Whitney *U* test. The categorical data for acceptable and unacceptable alignment were presented as counts and percentages. Differences in incidences of malalignment were analyzed using the Pearson’s Chi-square test. A *p* value under 0.05 was considered statistically significant.

## Results

### How accurate can surgeons estimate and correct tibia (mal)rotation according to present clinical standards?

The observers’ clinical judgment of the (mal)rotated injured limb with use of single-leg draping deviated 10.0° (IQR 15.0°) from the rotation that was applied. Subsequent correction, based on one leg being available to judge the reduction, resulted in a median malalignment of 10.0° (IQR 5.0°) (Table 1). A total of 33% of the attempts resulted in acceptable rotational alignment (<10°) whereas the remaining 67% resulted in an unacceptable alignment (>10°) (Table 1). In terms of the accuracy of clinical assessment, there was no difference between consultant surgeons and residents with regards to clinical judgment and correction for malalignment with use of single-leg draping (*p* = 0.89) (Table 2).

**Table 1:** The accuracy of correction for rotational malalignment according to present clinical standards and the CARV-protocol

	Single-leg draping	Dual-leg draping	CARV
Rotation relative to neutral alignment **(**median ± IQR)	10.0° (5.0°)	10.0° (5.0°)	5.0° (5.0°)
Acceptable alignment (<10°) (*n*, %)	20 (33%)	25 (42%)	53 (88%)
Unacceptable alignment (≥10°) (*n*, %) 10°–19° (*n*, %) 20°–29° (*n*, %) ≥30° (*n*, %)	40 (67%) 35 (60%) 5 (7%) 0 (0%)	35 (58%) 29 (48%) 5 (8%) 1 (2%)	7 (12%) 7 (12%) 0 (0%) 0 (0%)

**Table 2:** The relationship between level of experience and accuracy of correction for rotational malalignment using CARV, single-leg or dual-leg draping, respectively

Method	Correction		*p* value
	Consultant	Resident	
Single-leg draping (median ± IQR)	10.0º (6.3º)	10.0º (5.0º)	0.89
Dual-leg draping (median ± IQR)	10.0º (6.3º)	10.0º (5.0º)	0.98
CARV (median ± IQR)	5.0º (5.0º)	5.0º (5.0º)	0.73

Clinical estimation with use of dual-leg draping, allowing for direct comparison of the rotational profiles of both limbs, deviated 12.5° (IQR 15.0°) from the rotation that was applied. Subsequent correction, allowing for making the appearance of the legs identical, resulted in a median deviation of 10.0° (IQR 5.0°) from neutral alignment (Table 1). A total of 42% of the corrections represented acceptable rotational alignment (<10°). The remaining 58% accounted for unacceptable alignment (≥10°) (Table 1). We found no relationship between the level of experience and the accuracy of correction after dual-leg draping (*p* = 0.98) (Table 2).

### Can we improve the accuracy of rotational alignment by use of the CARV-protocol?

To answer the primary research question with regards to the accuracy of CARV, we found that correction with use of the CARV-protocol deviated at a median of 5.0° (IQR 5.0°) from neutral alignment (Table 1). A total of 88% of the attempts resulted in a rotation of <10° indicating acceptable alignment. Only 12% was categorized as an unacceptable alignment (≥10°) (Table 1). Both residents and consultants were able to apply the CARV-technique without differences in performance between groups (*p* = 0.73) (Table 2). Application of the CARV-protocol showed a significantly lower proportion of unacceptable rotational alignment compared to unstandardized correction with use of single-leg draping, (67 vs 12%, *p* < 0.001) and unstandardized correction with use of dual-leg draping (58 vs 12%, *p* < 0.001) (Table 3).

**Table 3:** The incidence of rotational malalignment after application of the CARV versus single-leg and dual-leg draping

	CARV	Single-leg draping	Dual-leg draping	CARV vs. single-leg draping	CARV vs. dual-leg draping
Acceptable (<10º)	53 (88%)	20 (33%)	25 (42%)	–	–
Unacceptable (≥10º)	7 (12%)	40 (67%)	35 (58%)	–	–
				*p* < 0.001	*p* < 0.001
Total observations	60	60	60	–	–

### Clinical feasibility of the CARV-protocol

A total of three consecutive patients (43 years, 43 years, 19 years) underwent intramedullary-nailing with application of the CARV-protocol. Different fracture patterns were included (AO/OTA [28] type 42-B2, 42-C2, 42-B3). We were able to apply the CARV-protocol clinically as proposed in our experimental study setup. The clinical cases demonstrate the feasibility of CARV in clinical practice. The rotational outcomes based on postoperative CT-assessment are presented in the supplementary materials. The rotational alignment in cases 1–3 were, respectively, 3°, 4° and 8°, indicating acceptable tibial alignment in all cases.

## Discussion

There is a high incidence of rotational malalignment following intramedullary-nailing for tibia shaft fractures with incidences up to 30% [1–7]. An intraoperative fluoroscopy protocol to increase accuracy of alignment control during intramedullary-nailing of these fractures is still lacking. This study is the first to present an accurate and clinically feasible standardized intraoperative fluoroscopy protocol coined ‘C-Arm Rotational View (CARV)’ in order to minimize the risk on rotational malalignment, and to avoid rotational outliers during intramedullary-nailing of tibia shaft fractures.

Our primary findings demonstrate that tibial alignment during intramedullary-nailing of tibia shaft fractures can be significantly improved using the CARV-protocol. Only 12% of the corrections were categorized as unacceptable rotational alignment (≥10°). Second, we found that clinical estimation of rotation followed by realignment of the tibia according to present clinical standards is inaccurate. A total of 67% of the rotational corrections after single-leg draping and 58% after dual-leg draping resulted in unacceptable rotational alignment (≥10°). Application of the CARV relative to current clinical practice decreased the rate of rotational alignment from 67 and 58% to 12%, respectively (*p* < 0.001).

This study should be interpreted considering strengths and weaknesses. The CARV-protocol was tested in cadaveric specimens in which only a transverse mid-shaft fractures was simulated instead of different fracture patterns. We felt that incorporating a mid-shaft fracture in the study setup allowed for adequate measuring of tibia (mal)alignment using reference wires on both fracture sites. We believe that the simplified nature of our test setup does not disqualify our findings. Moreover, CARV was successfully applied in the case series that included different fracture patterns. Although proven to be clinically feasible, prospective clinical studies are needed to clinically validate the CARV-protocol on a larger scale. Secondly, the CARV-method works under assumption that each individual has almost symmetric tibias leading to symmetric radiographic landmarks. A previous study by our group demonstrated a potential physiological difference of 4° between the right and left tibiae [7]. We believe that the small difference between the right and left tibiae does not have compromised the performance of CARV-protocol in this experimental study.

In femoral shaft fractures, multiple simple intraoperative fluoroscopy protocols have been described to avoid rotational malalignment after intramedullary-nailing [20–24]. Similarly, the potential of rotational malalignment following intramedullary-nailing for tibia shaft fractures can be minimized by simple application of the CARV-protocol. Some methods for avoiding rotational malalignment have been described in literature. Recently, a case report reported on the perfect lateral view of the ankle in order to obtain adequate alignment during intramedullary-nailing for tibia shaft fractures [18]. However, this technique was not tested or validated in a research setting confirming its accuracy and usability. Clementz et al. [16] introduced a fluoroscopy-technique in 1989 measuring the angle between femur and ankle by assessing the overlap of the anterior and posterior cortex of the medial malleolus. This technique did not find its way in standard clinical practice despite multiple attempts to endorse its feasibility [29, 30].

Correction according to present clinical standards using either single-leg or dual-leg seemed to be insufficient in this experimental study, and has proven insufficient in multiple clinical prospective cohort studies [1–7]. This inaccuracy may be caused by (1) difficulties in clinical estimation and (2) the absence of a standardized fluoroscopy protocol to obtain adequate alignment. Among observers, the cortical step sign (CSS) and diameter difference sign (DDS) was used for alignment control [17]. Although Keppler et al. [19] proved the CSS and DSS to be reliable landmarks to detect and correct for malrotation, we feel that the clinical feasibility is limited due to differences in fracture patterns and possible axial translation of the tibia caused by the eccentric position of the intramedullary-nail in the tibia shaft.

The first advantage of the CARV-protocol was the use of the C-arm as simple and accurate indicator for rotational malalignment rather than inaccurate clinical judgment. The C-arm was able to detect a 5° rotational difference between both injured and uninjured limb by revealing sufficient small fluoroscopy alterations of the proximal tibiofibular overlap and mortise-view of the ankle (Fig. 2). A second asset of the CARV-protocol includes the simple standardization of rotational correction techniques. The need for such was strengthened by the existence of a wide range of insufficient correction techniques among observers which resulted in relatively high rates of unacceptable rotational outcomes. We found no relationship between level of experience and successful application of the CARV-technique, and finally, the CARV-protocol has proven to be accurate in a clinical setting with different fracture patterns underlying its potential practicability and reproducibility in clinical practice.

## Conclusion

This experimental study is one of the first to present a simple standardized intraoperative fluoroscopy protocol named the ‘C-arm Rotational View (CARV)’ to reduce iatrogenic rotational malalignment following intramedullary-nailing for patients with a tibia shaft fracture. The CARV-protocol has proven accurate and reproducible in cadaveric specimens and feasible in several clinical cases. Both consultants and residents successfully applied the CARV-protocol. Future prospective cohort studies are needed to determine the diagnostic performance characteristics in clinical practice.

### Supplementary Information

Below is the link to the electronic supplementary material.

Supplementary file1 (TIF 21338 KB)

Supplementary file2 (TIF 24326 KB)

Supplementary file3 (TIF 36343 KB)

Supplementary file4 (TIF 23161 KB)

Supplementary file5 (TIF 30354 KB)

Supplementary file6 (TIF 48337 KB)

Supplementary file7 (TIF 9176 KB)

Supplementary file8 (TIF 28161 KB)

Supplementary file9 (TIF 40205 KB)

Supplementary file10 (DOCX 20 KB)
