# Correction: Intraoperative fluoroscopic protocol to avoid rotational malalignment after nailing of tibia shaft fractures: introduction of the ‘C-Arm Rotational View (CARV)’

**DOI:** 10.1007/s00068-022-02098-4

**Published:** 2022-11-21

**Authors:** Nils Jan Bleeker, Job N. Doornberg, Kaj ten Duis, Mostafa El Moumni, Inge H. F. Reininga, Ruurd L. Jaarsma, Frank F. A. IJpma, On behalf of the Traumaplatform 3D Consortium, L. M. Goedhart, B. de Cort, L. A. M. Hendrickx, M. ter Horst, J. Gorter, R. J. van Luit, P. Nieboer, W. Füssenich, T. Zwerver, R. Koster, J. J. Valk, L. Reinke, J. G. Bleeker, M. Cain, F. J. P. Beeres, G. M. M. J. Kerkhoffs

**Affiliations:** 1 Department of Orthopaedic Trauma Surgery, University Medical Center Groningen, University of Groningen, Groningen, The Netherlands; 2 Department of Orthopaedic Trauma Surgery, Flinders Medical Center and Flinders University, Adelaide, Australia

## Correction: European Journal of Trauma and Emergency Surgery 10.1007/s00068-022-02038-2

In the Acknowledgements section the following part was missing: On behalf of the Traumaplatform 3D Consortium: L. M. Goedhart, B. de Cort, L. A. M. Hendrickx, M. ter Horst, J. Gorter, R. J. van Luit, P. Nieboer, W. Füssenich, T. Zwerver, R. Koster, J. J. Valk, L. Reinke, J. G. Bleeker, M. Cain, F. J. P. Beeres, G. M. M. J. Kerkhoffs.

The original article has been corrected.

