# Supplementary figures and images for: Intraoperative fluoroscopic protocol to avoid rotational malalignment after nailing of tibia shaft fractures: introduction of the ‘C-Arm Rotational View (CARV)’
Source: Eur J Trauma Emerg Surg. 2022 Jul 30;49(6):2329–36. doi: 10.1007/s00068-022-02038-2 (PMC10728226; doi:10.1007/s00068-022-02038-2)

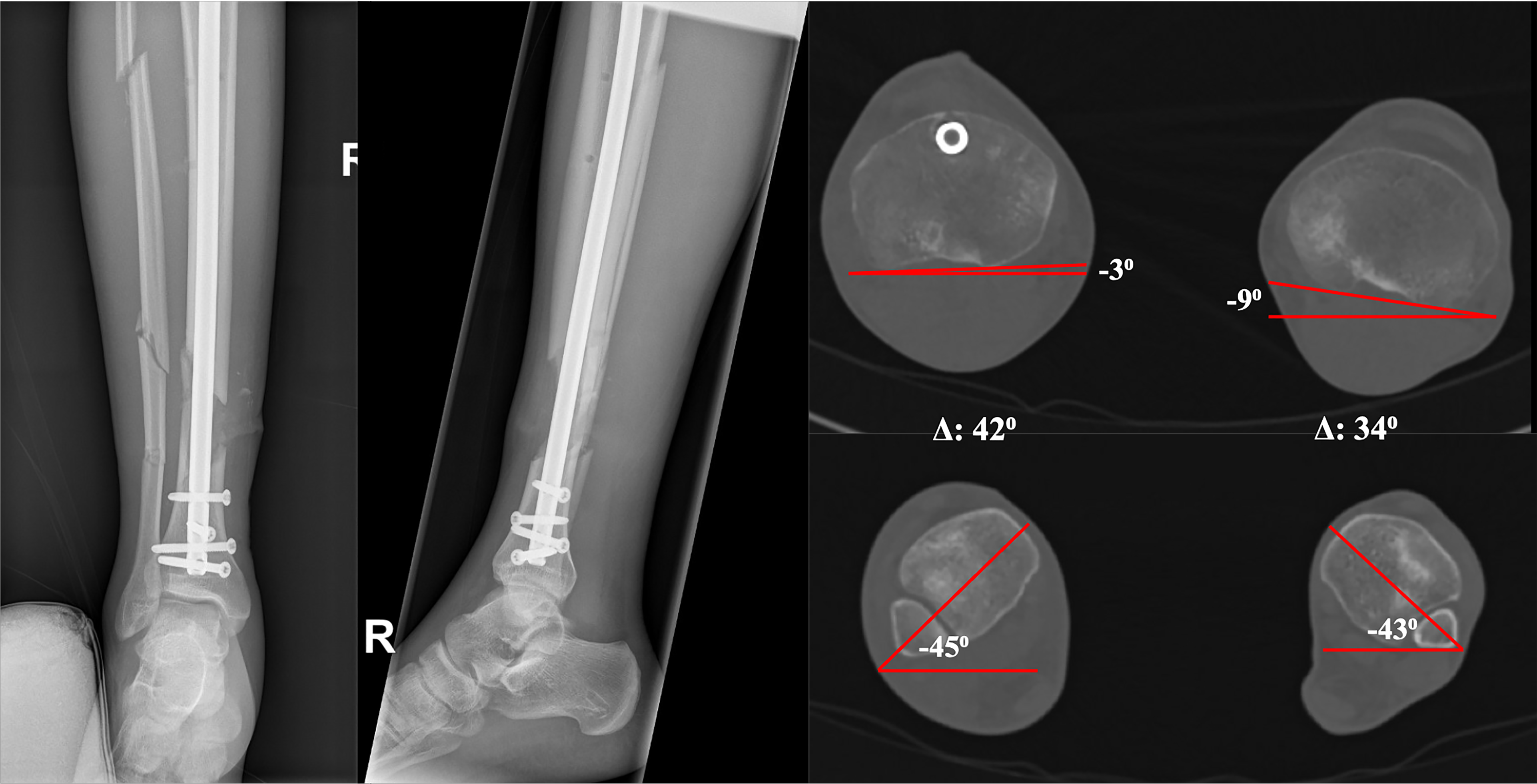

Supplement: Supplementary file 1 — Supplementary file1 (TIF 21338 KB) [file 68_2022_2038_MOESM1_ESM.tif]

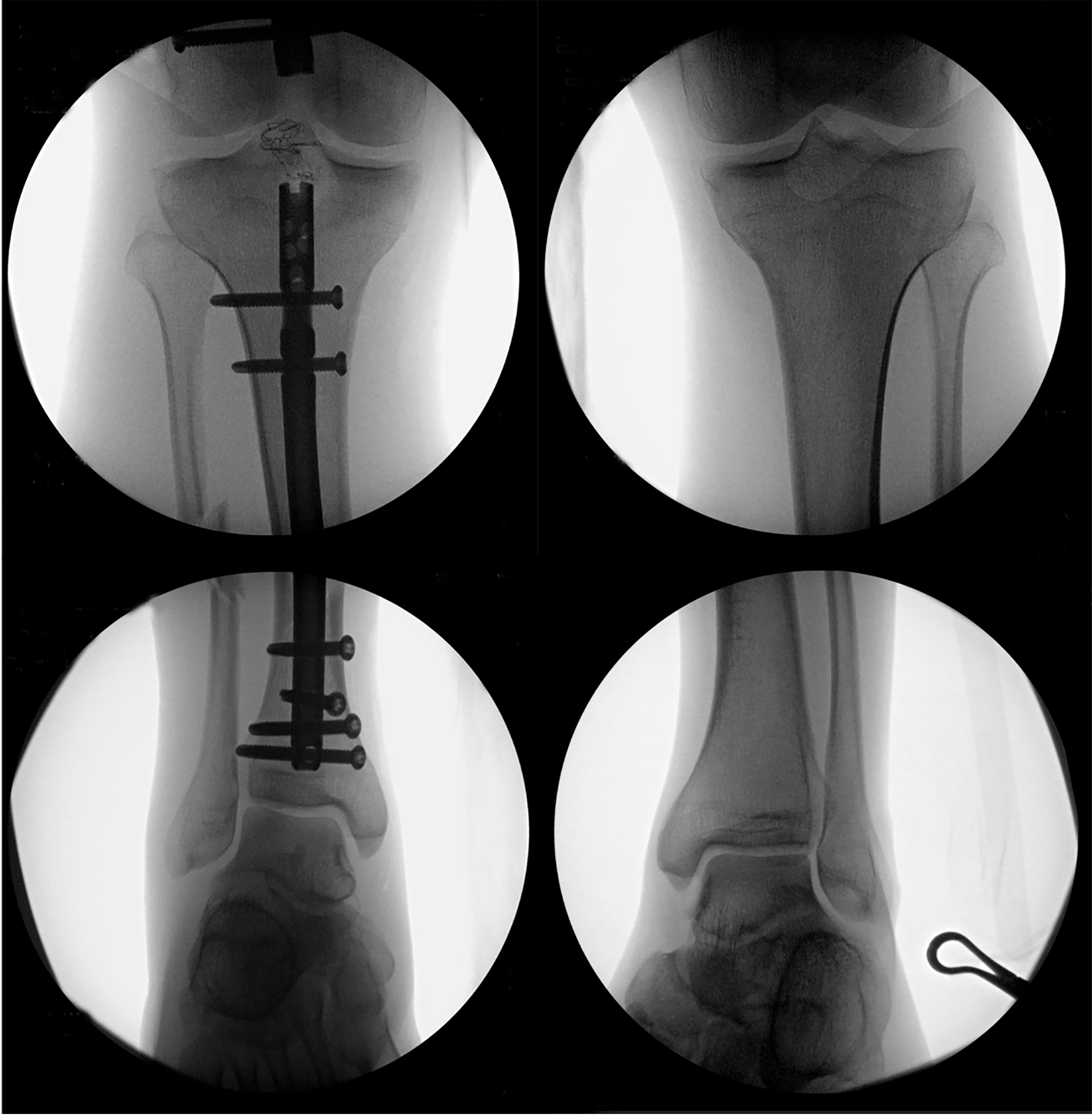

Supplement: Supplementary file 2 — Supplementary file2 (TIF 24326 KB) [file 68_2022_2038_MOESM2_ESM.tif]

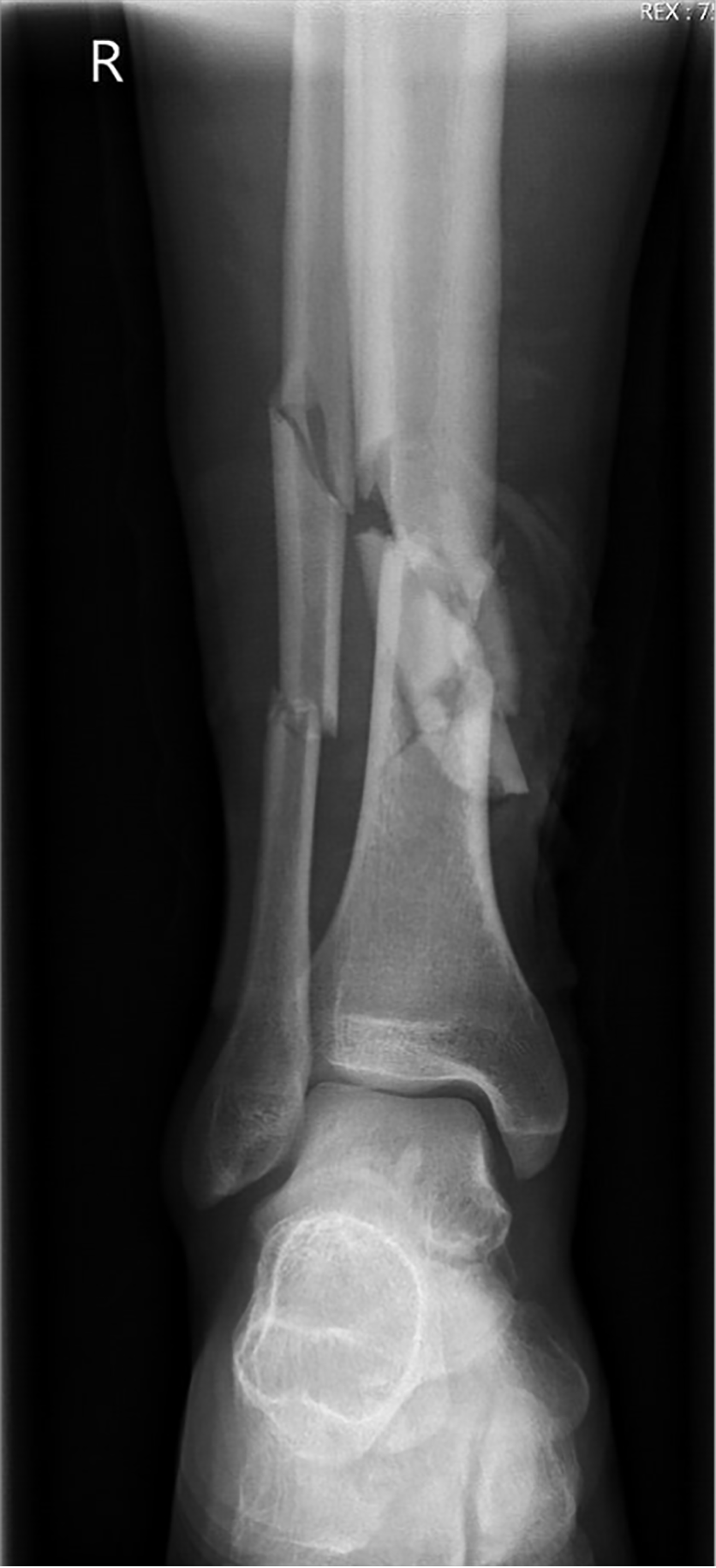

Supplement: Supplementary file 3 — Supplementary file3 (TIF 36343 KB) [file 68_2022_2038_MOESM3_ESM.tif]

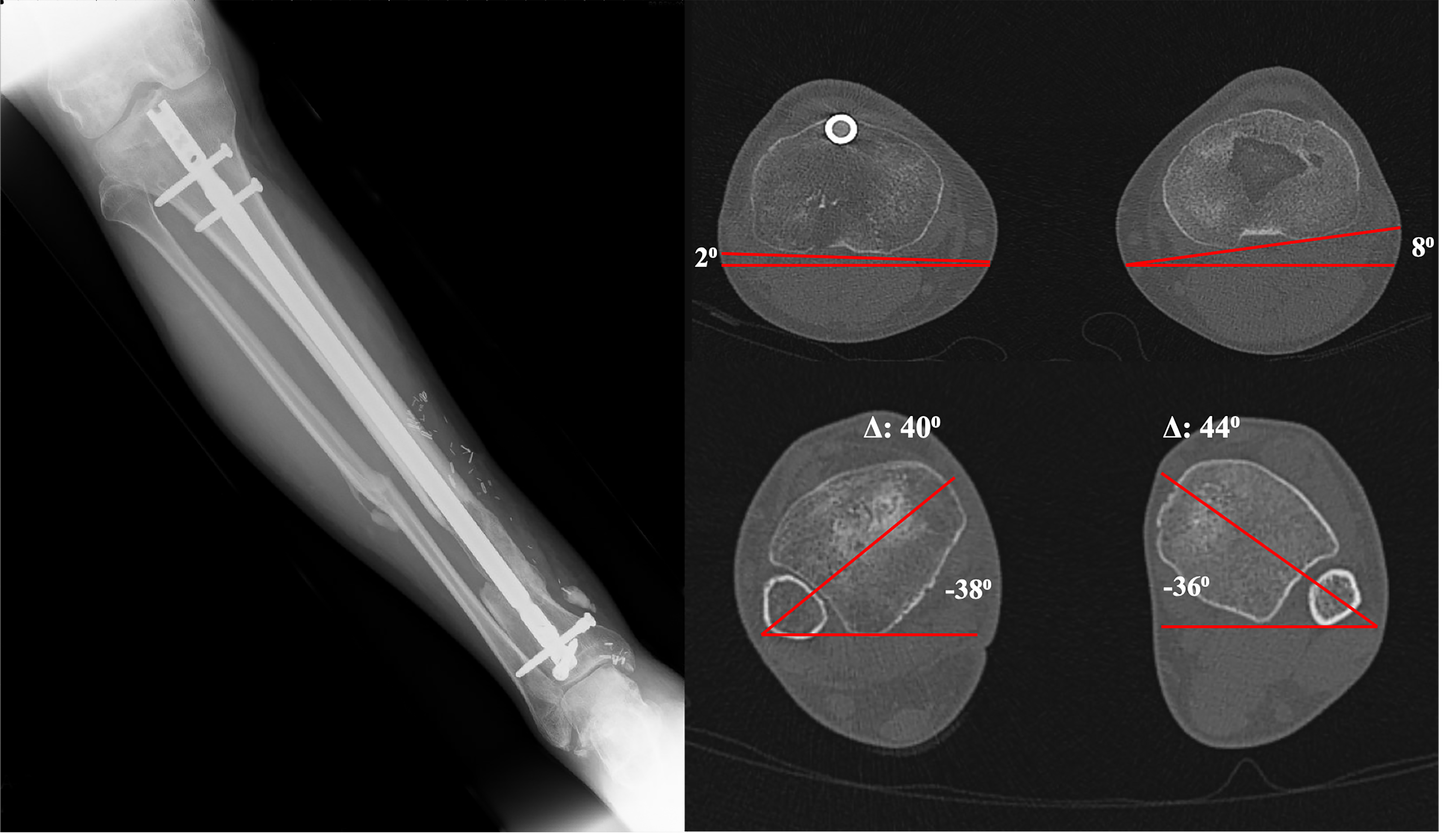

Supplement: Supplementary file 4 — Supplementary file4 (TIF 23161 KB) [file 68_2022_2038_MOESM4_ESM.tif]

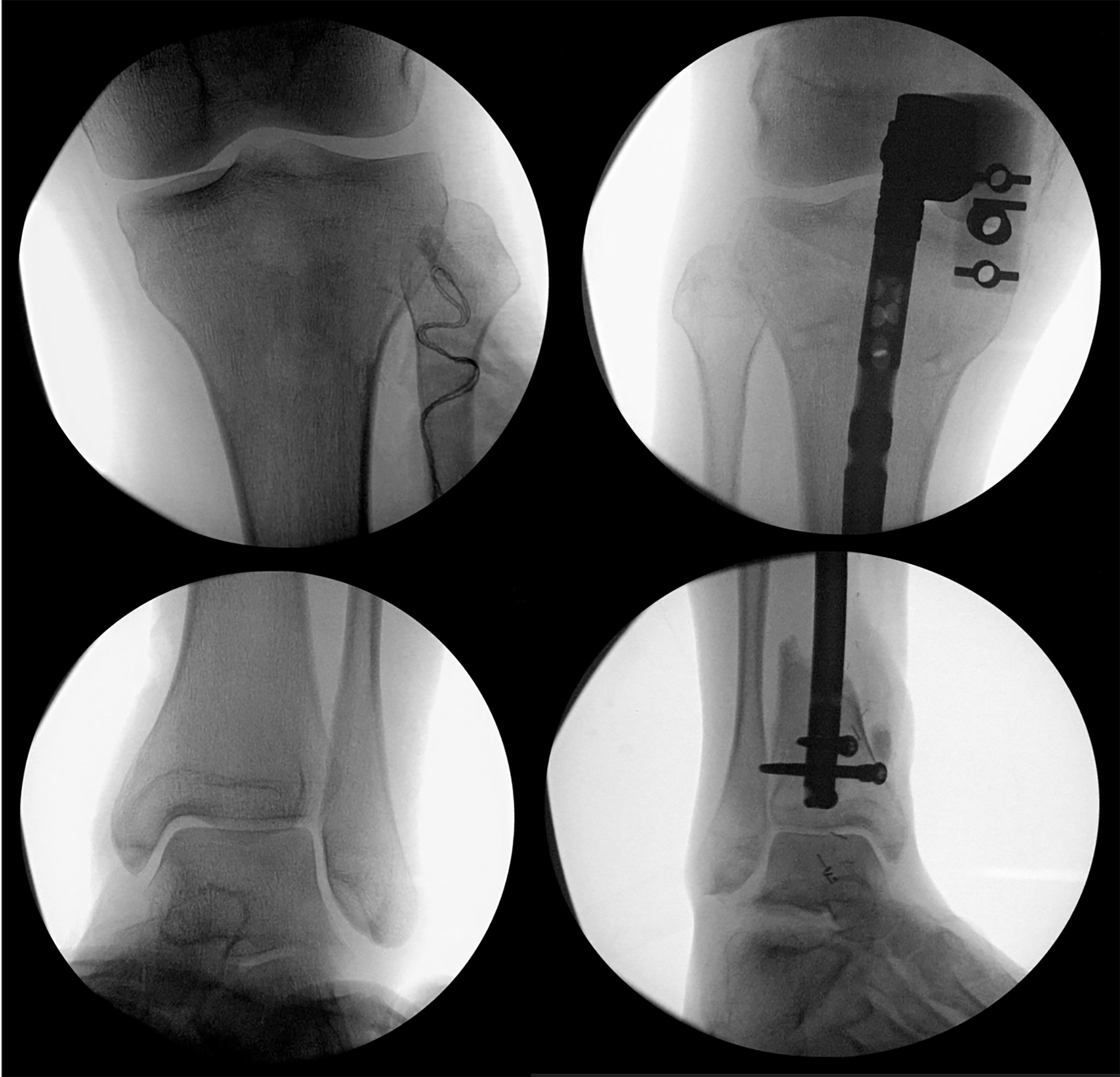

Supplement: Supplementary file 5 — Supplementary file5 (TIF 30354 KB) [file 68_2022_2038_MOESM5_ESM.tif]

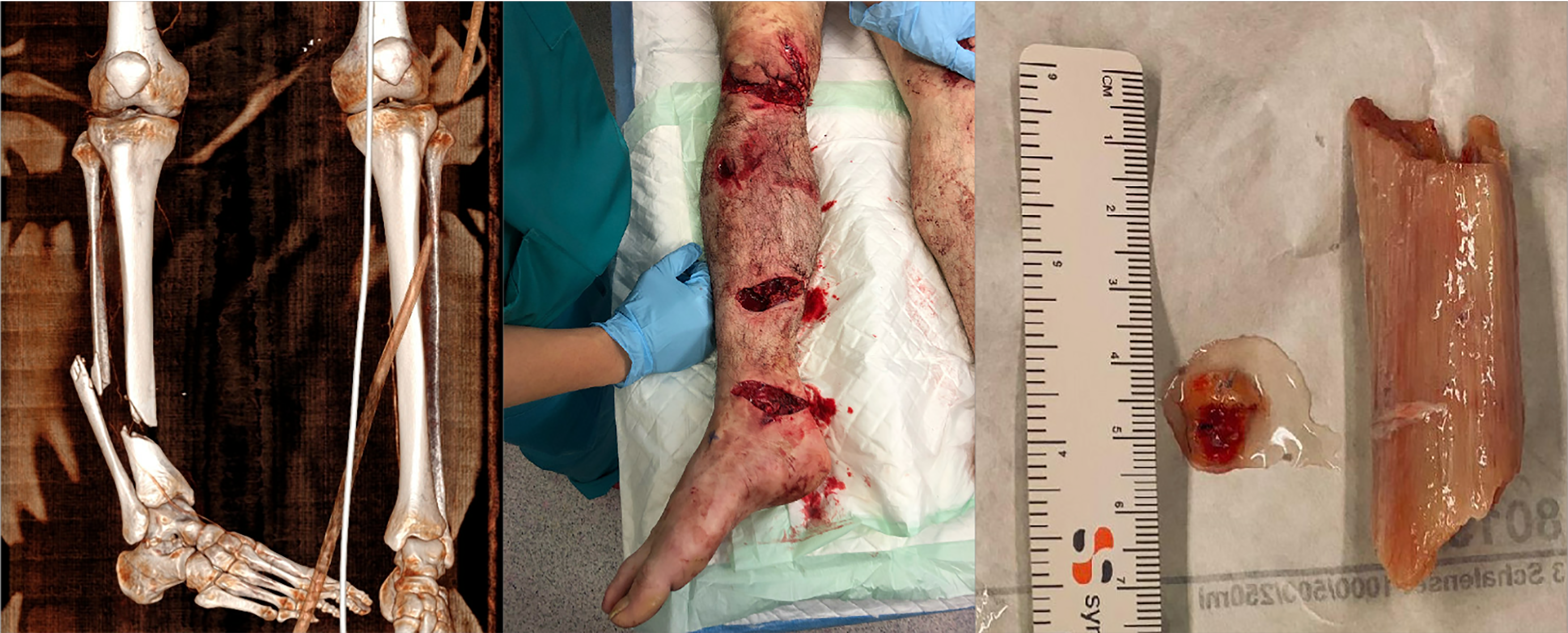

Supplement: Supplementary file 6 — Supplementary file6 (TIF 48337 KB) [file 68_2022_2038_MOESM6_ESM.tif]

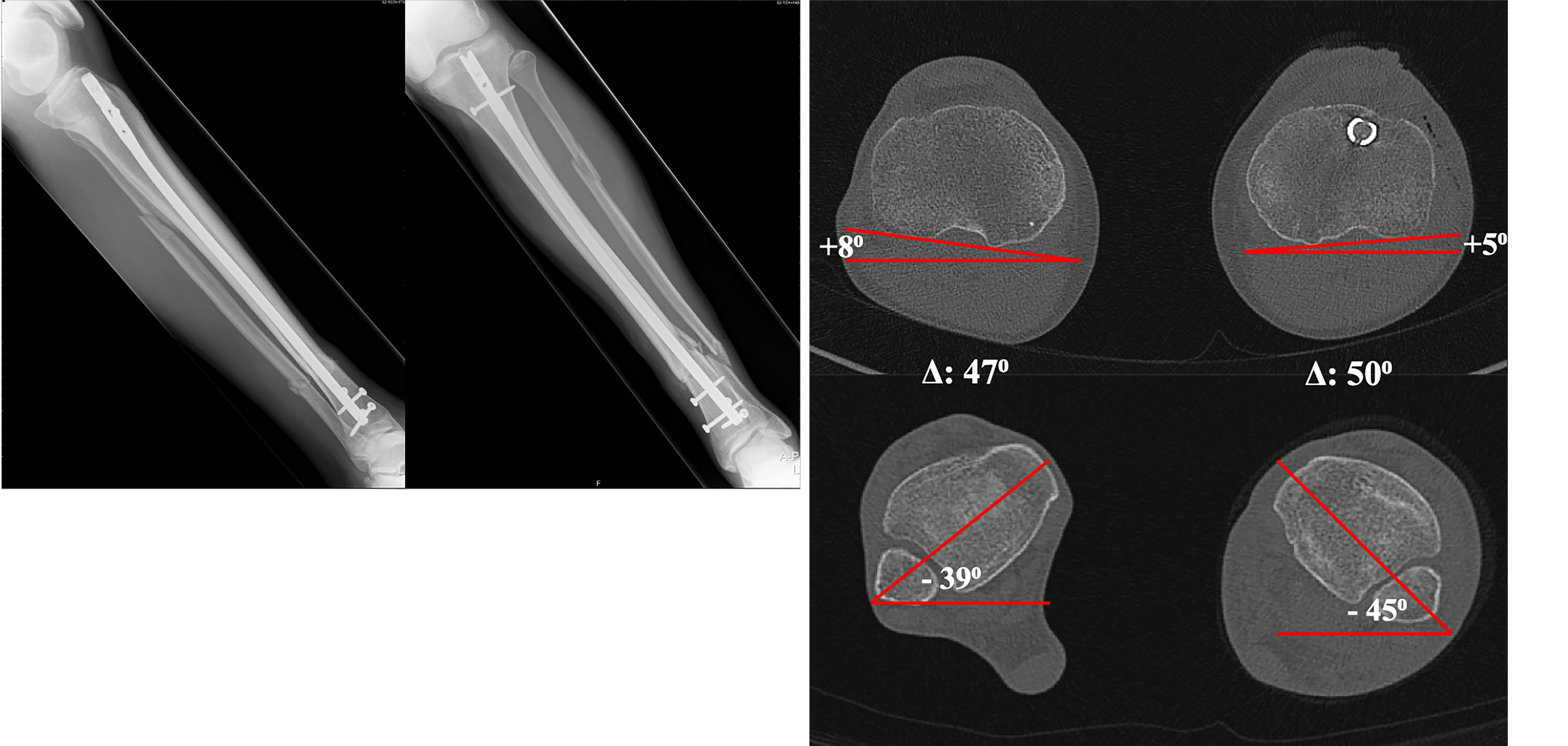

Supplement: Supplementary file 7 — Supplementary file7 (TIF 9176 KB) [file 68_2022_2038_MOESM7_ESM.tif]

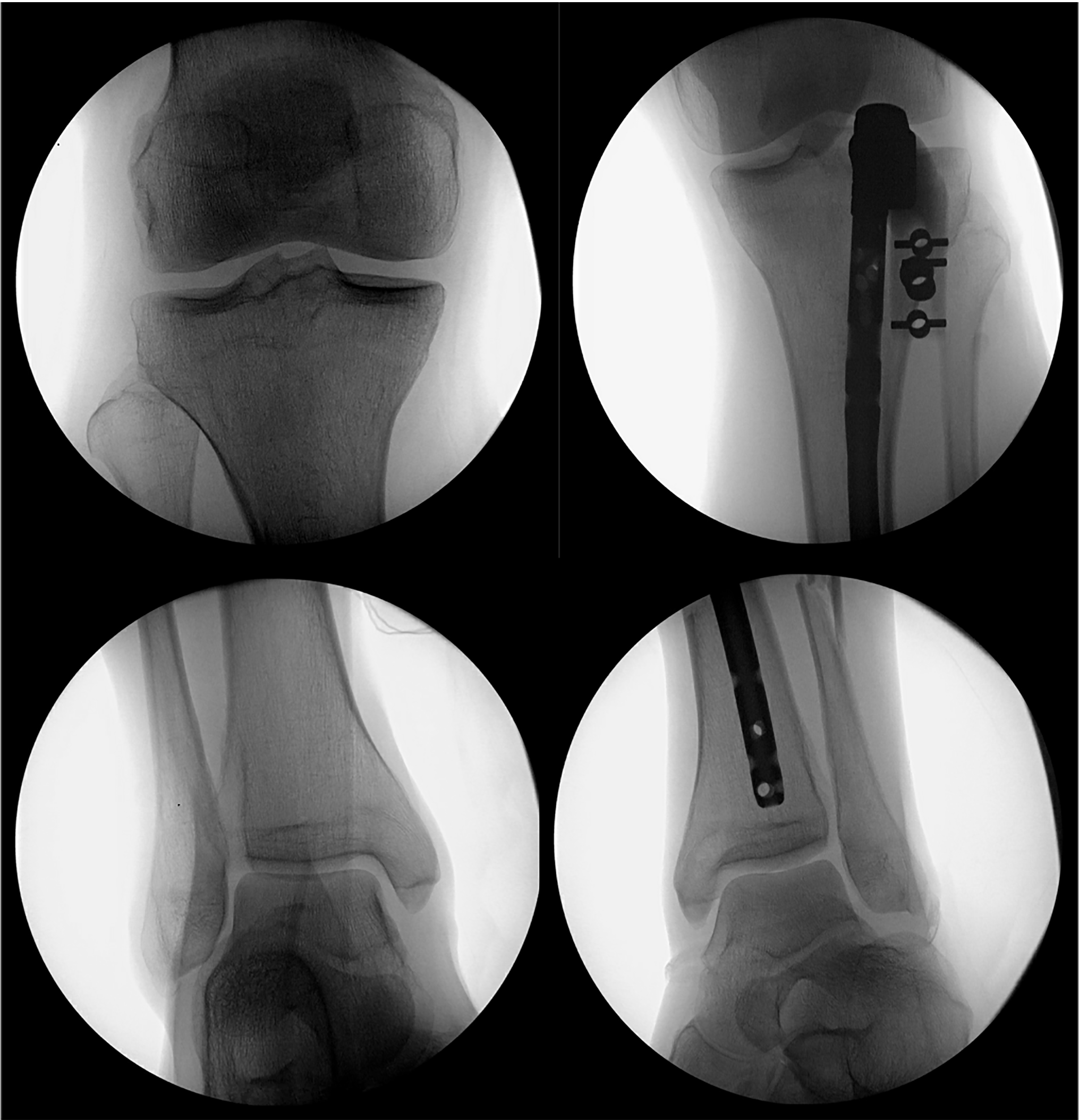

Supplement: Supplementary file 8 — Supplementary file8 (TIF 28161 KB) [file 68_2022_2038_MOESM8_ESM.tif]

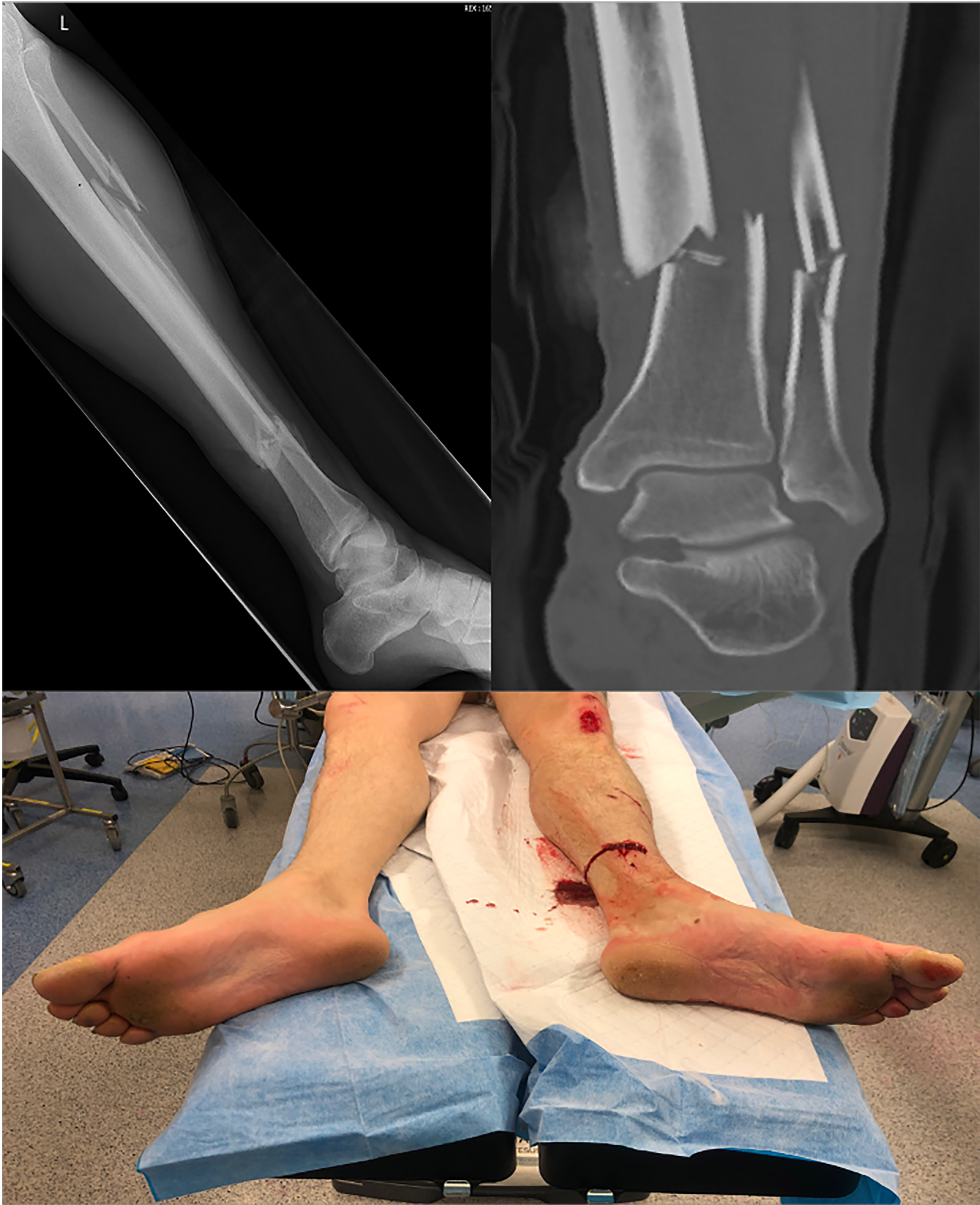

Supplement: Supplementary file 9 — Supplementary file9 (TIF 40205 KB) [file 68_2022_2038_MOESM9_ESM.tif]
