# Supplementary material for: Intraoperative fluoroscopic protocol to avoid rotational malalignment after nailing of tibia shaft fractures: introduction of the ‘C-Arm Rotational View (CARV)’
Source: Eur J Trauma Emerg Surg. 2022 Jul 30;49(6):2329–36. doi: 10.1007/s00068-022-02038-2 (PMC10728226; doi:10.1007/s00068-022-02038-2)
Supplement: Supplementary file 10 — Supplementary file10 (DOCX 20 KB) [file 68_2022_2038_MOESM10_ESM.docx]

**Supplementary data file 1: clinical cases.**

**Case 1:** Male, 43 years, left sided tibia fracture.

**Injury mechanism:** motorcycle accident.

**Preoperative radiographs / clinical impression:** tibia fracture, AO/OTA 42-B2.

(Appendix figure 1)

**Operation:** Intramedullary-nailing with C-Arm Rotational View (CARV)

(Appendix figure 2)

**Postoperative radiographs and CT-assessment:**

(Appendix figure 3)

**Appendix figure 3 legend:** The rotational (mal)alignment is the rotational difference between the injured- and uninjured side. In this case, the rotation of the injured side is 50° (- 45° - 5°). The rotation of the uninjured side is 47° (- 39° - 8°). The rotation is thus 3° (injured side (50°) – uninjured side (47°)) and indicated *acceptable* alignment.

**Case 2:** Male, 43 years, with a Gustilo grade 3 open right sided tibia fracture and a segmental defect of the tibia.

**Injury mechanism:** car accident, polytrauma.

**Pre-operative CT / clinical impression:** tibia fracture with a 7 cm segmental defect, AO/OTA 42-C2.

(Appendix figure 4)

**Operation 1**: temporary external fixator, cement spacer, radialis flap.

**Operation 2:** intramedullary-nailing with C-Arm Rotational View (CARV) and cancellous bone grafting.

(Appendix figure 5)

**Postoperative radiographs and CT-assessment:**

(Appendix figure 6)

**Appendix figure 6 legend:** The rotational (mal)alignment is the rotational difference between the injured- and uninjured side. In this case, the rotation of the injured side is 40° (- 38° - 2°). The rotation of the uninjured side is 44° (- 36° - 8°). The rotation is thus 4° (injured side (40°) – uninjured side (44°)) and indicated *acceptable* alignment.

**Case 3:** Male, 19 years, with a right sided comminuted tibia fracture.

**Injury mechanism**: motorcycle accident.

**Pre-operative radiographs:** tibia shaft fracture, AO/OTA 42-B3.

(Appendix figure 7)

**Operation:** intramedullary-nailing with C-arm Rotational View (CARV).

(Appendix figure 8)

**Postoperative radiographs and CT-assessment:**

(Appendix figure 9)

**Appendix figure 9 legend:** The rotational (mal)alignment is the rotational difference between the injured- and uninjured side. In this case, the rotation of the injured side is 42° (- 45° - -3°). The rotation of the uninjured side is 34° (- 43° - -9°). The rotation is thus 8° (injured side (42°) – uninjured side (34°)) and indicated *acceptable* alignment.
